# Dual delivery of antigens shows promise

**DOI:** 10.7554/eLife.90407

**Published:** 2023-07-21

**Authors:** Jose L Luque-García, Rafael Prados-Rosales

**Affiliations:** 1 Department of Analytical Chemistry, School of Chemistry, Universidad Complutense of Madrid Madrid Spain; 2 Department of Preventive Medicine, Public Health and Microbiology, School of Medicine, Universidad Autonoma de Madrid Madrid Spain

**Keywords:** tuberculosis, vaccines, mycolic acids, T cells, mycobacterial antigens, Mycobacterium tuberculosis, Mouse

## Abstract

The simultaneous delivery of protein and lipid antigens via nanoparticles may help efforts to develop a new vaccine for tuberculosis.

**Related research article** Morgun E, Zhu J, Almunif S, Bobbala S, Aguilar MS, Wang J, Conner K, Cui Y, Cao L, Seshadri C, Scott EA, Wang C. 2023. Vaccination with mycobacterial lipid loaded nanoparticle leads to lipid antigen persistence and memory differentiation of antigen-specific T cells. *eLife* **12**:RP87431. doi: 10.7554/eLife.87431.

The BCG vaccine was first used in 1921 and, remarkably, over 100 years later it is the only licensed vaccine for use against tuberculosis. While the BCG vaccine provides protection against tuberculosis that has spread beyond the lungs in young children, its efficacy against lung infections in adults – the main form of the disease – is variable (Trunz et al., 2006). Tuberculosis is caused by a bacterium called *Mycobacterium tuberculosis* (*M. tb*), and one reason for the lack of more effective vaccines is that we still do not know which *M. tb* antigens are able to induce protective immunity (Guinn and Rubin, 2017).

Almost 20 vaccine candidates are currently being evaluated in clinical trials. Most of these aim to induce a response from immune cells called type 1 helper T cells by exposing them to protein antigens from *M. tb*, although other approaches are also being explored. Now, in eLife, Evan Scott and Chyung-Ru Wang from Northwestern University and colleagues – including Eva Morgun as first author – report a new approach that involves using protein antigens and long-chain fatty acids called mycolic acids, found in the *M. tb* cell envelope, to induce an immune response (Morgun et al., 2023).

Previous studies have shown that the immune system generates a protective response when exposed to mycolic acids: this response is mediated by a subset of “unconventional” T cells, but the details of the response are not fully understood (Shang et al., 2018; Zhao et al., 2015). Moreover, there have been very few attempts to explore the use of mycolic acids as alternative antigens for a tuberculosis vaccine, partly because most vaccine delivery systems are designed for hydrophilic cargoes (such as proteins), whereas mycolic acids are hydrophobic. Morgun et al. have overcome this problem by developing a system that can deliver hydrophobic and hydrophilic cargoes at the same time.

Building upon evidence that vaccine formulations including *M. tb* lipids encapsulated in liposomes – hollow spherical structures made of lipid bilayers – provide protection (Dascher et al., 2003; Larrouy-Maumus et al., 2017), Morgun et al. developed structures called bicontinuous nanospheres. These self-assembled polymer-based structures can carry both hydrophobic and hydrophilic components because they possess an organized internal cubic structure including hydrophobic bilayers and hydrophilic aqueous channels (Figure 1). Furthermore, the nanospheres retain antigens more efficiently than other lipid nanostructures, and only release their contents when they reach the target compartment within antigen-presenting cells. These features increase the persistence of the antigens within cells and stimulate the immune system for longer periods of time, which leads to a more robust immune response (Correia-Pinto et al., 2013).

**Figure 1.**
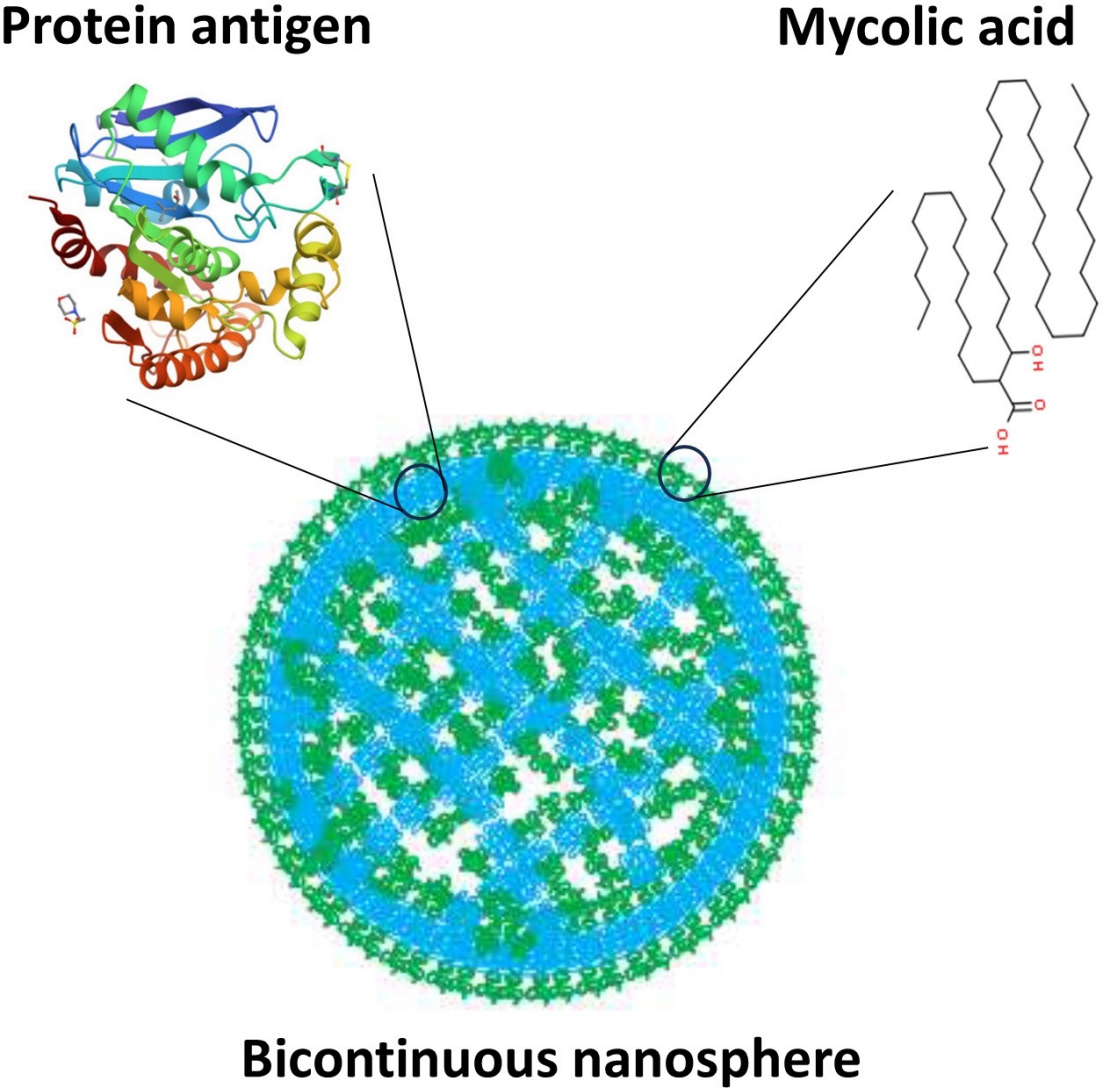
Bicontinuous nanospheres can be loaded with both protein and lipid antigens. Structure of a bicontinuous nanosphere. Hydrophilic (blue) regions contain protein antigens and hydrophobic (green) regions contain mycolic acids, represented by tentative structures. Figure based on image from Allen et al., 2018.

Morgun et al. used a mouse model that allowed them to observe the immune response to dual vaccination with mycolic acids and proteins. This showed that T cells responded to both antigens: however, only mycolic acid remained detectable inside immune cells after vaccination. It remains unclear if this differential antigen persistence is due to the nanosphere delivery system, the protein itself, or a distinct mechanism of antigen capture and maintenance by immune cells.

It is worth noting that the persistence of mycolic acids likely occurs during in vivo infection and, therefore, the dual vaccination strategy may mimic a previously overlooked feature of the infection process. Supporting this notion, Morgun et al. found that vaccination with an attenuated *M. tb* strain resulted in a persistence of mycolic acids similar to that observed with the dual approach. These results suggest that vaccines using live attenuated strains of *M. tb* likely provide protection via mycolic acid-specific T cells. This raises the question of whether the persistence of lipid antigens is a key aspect of the protective immune response: this is something that needs to be considered when developing tuberculosis vaccines in the future.

Although definitive proof of protective immunity induced by dual-loaded bicontinuous nanospheres remains elusive, this approach allowing combinations of antigens – both hydrophobic and hydrophilic – can be used as an alternative strategy to live attenuated vaccines. Moreover, the ability to test combinations of alternative antigens will aid the development of vaccines for tuberculosis more generally. Future work with this system could also investigate the role of lipid persistence in other overlooked aspects of tuberculosis immunity, such as antibody responses, which might have an important contribution to protective immunity.
